# Supplementary figures and images for: Interaction of fungal lipase with potential phytotherapeutics
Source: PLoS One. 2022 May 26;17(5):e0264460. doi: 10.1371/journal.pone.0264460 (PMC9135303; doi:10.1371/journal.pone.0264460)

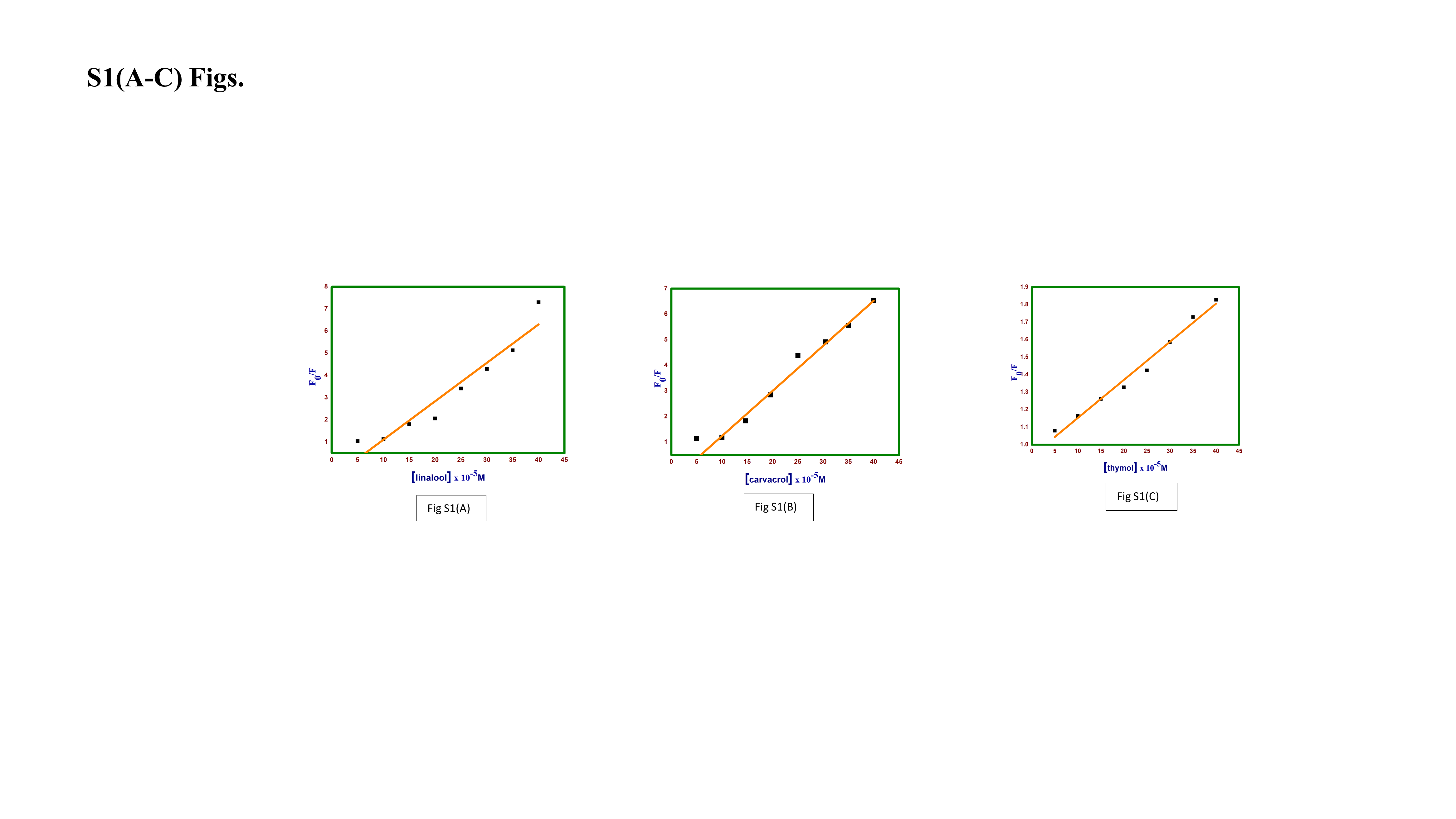

Supplement: S1 Fig — (TIF) [file pone.0264460.s001.tif]

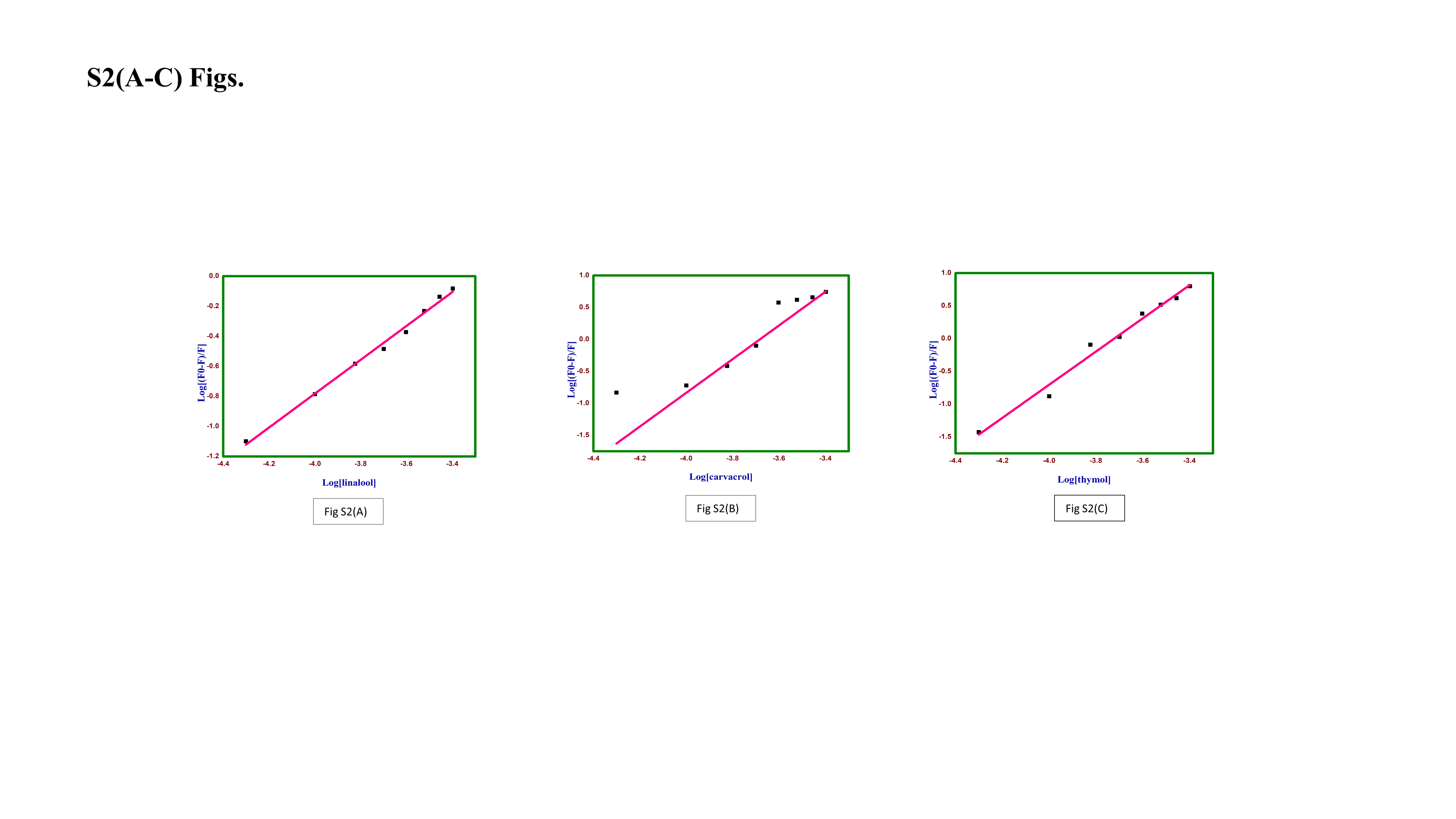

Supplement: S2 Fig — (TIF) [file pone.0264460.s002.tif]

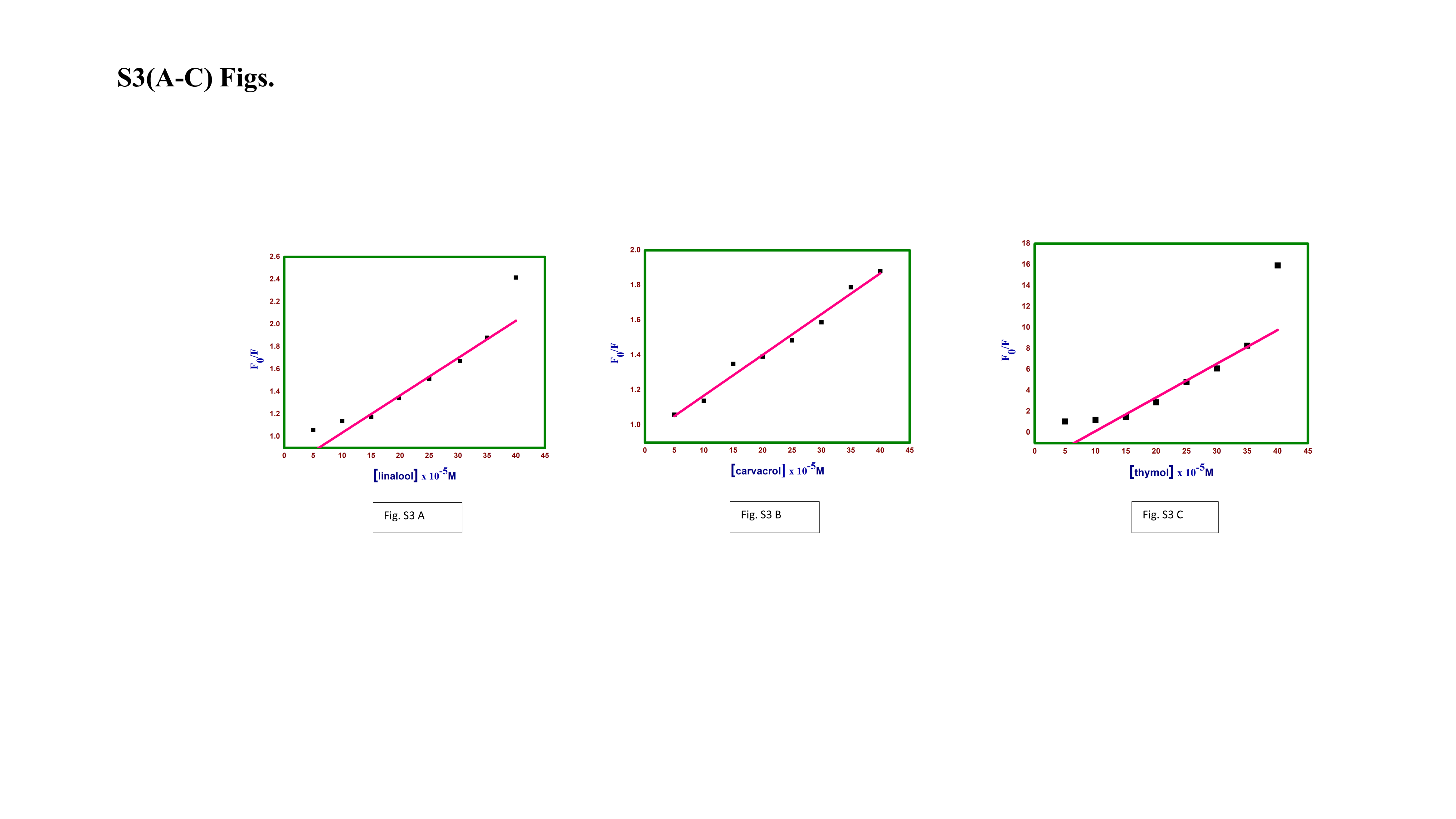

Supplement: S3 Fig — (TIF) [file pone.0264460.s003.tif]

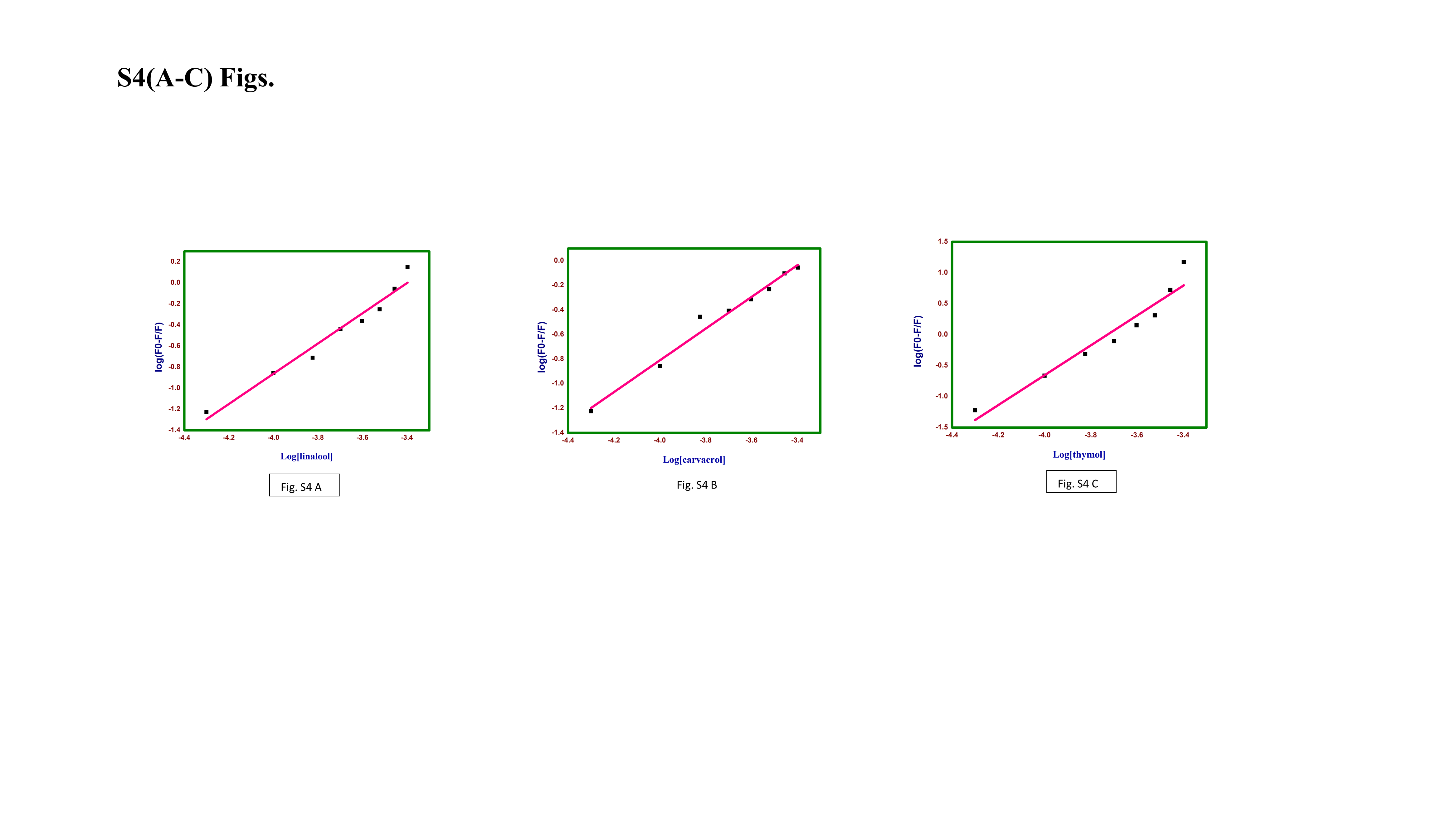

Supplement: S4 Fig — (TIF) [file pone.0264460.s004.tif]

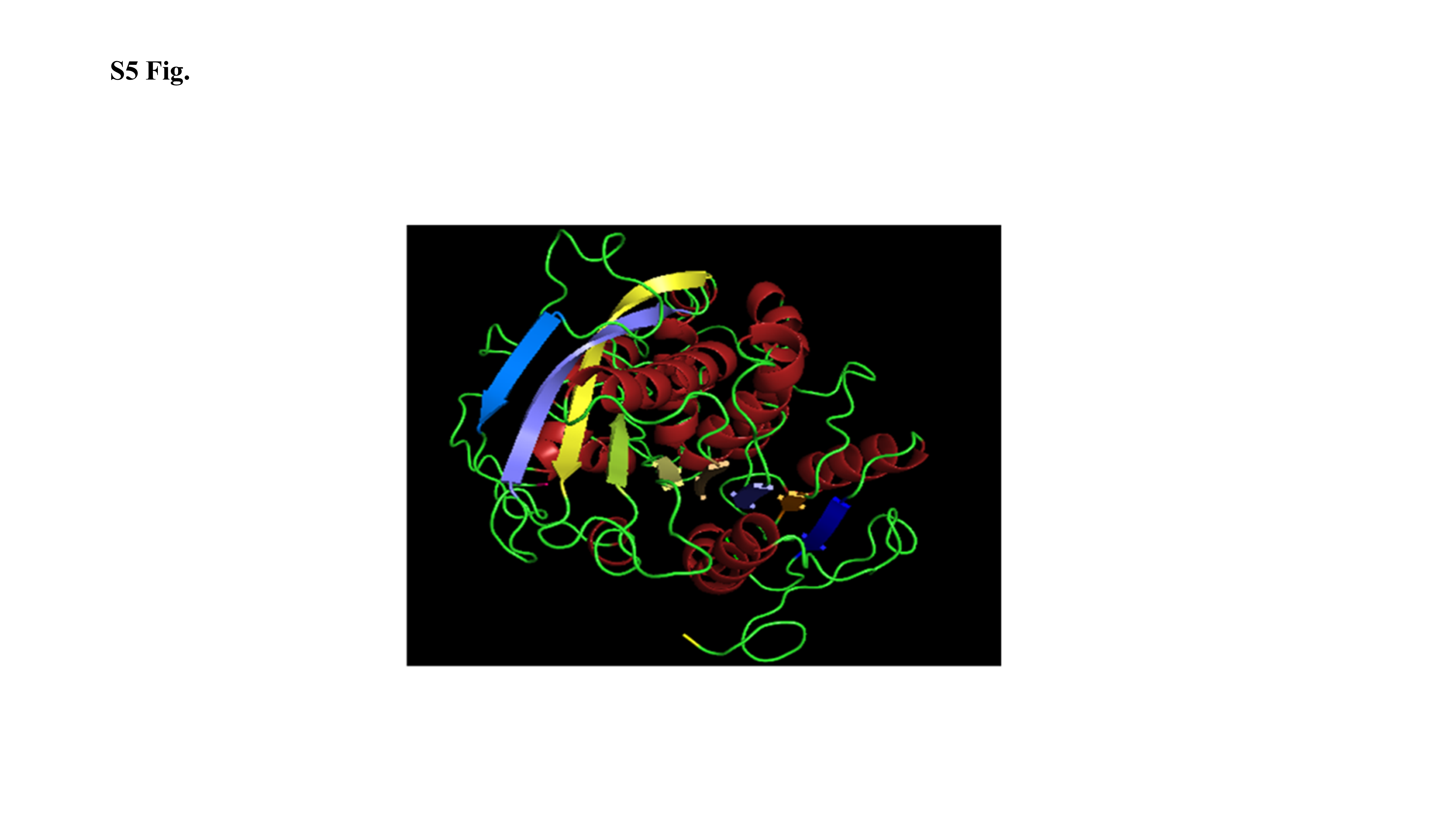

Supplement: S5 Fig — (TIF) [file pone.0264460.s005.tif]
